# The Reason for Growth Inhibition of *Ulmus pumila* ‘Jinye’: Lower Resistance and Abnormal Development of Chloroplasts Slow Down the Accumulation of Energy

**DOI:** 10.3390/ijms20174227

**Published:** 2019-08-29

**Authors:** Lihui Zuo, Shuang Zhang, Yichao Liu, Yinran Huang, Minsheng Yang, Jinmao Wang

**Affiliations:** 1 Institute of Forest Biotechnology, Forestry College, Agricultural University of Hebei, Baoding 071000, China; 2 Hebei Key Laboratory for Tree Genetic Resources and Forest Protection, Baoding 071000, China; 3 College of Landscape and Ecological Engineering, Hebei University of Engineering, Handan 056000, China; 4 Hebei Forestry Research Institute, Shijiazhuang 050000, China

**Keywords:** *Ulmus pumila* L., leaf color mutant, RNA seq, thylakoid

## Abstract

*Ulmus pumila* ‘Jinye’, the colorful leaf mutant of *Ulmus* *pumila* L., is widely used in landscaping. In common with most leaf color mutants, *U*. *pumila* ‘Jinye’ exhibits growth inhibition. In this study, *U.* *pumila* L. and *U*. *pumila* ‘Jinye’ were used to elucidate the reasons for growth inhibition at the physiological, cellular microstructural, and transcriptional levels. The results showed that the pigment (chlorophyll a, chlorophyll b, and carotenoids) content of *U.* *pumila* L. was higher than that of *U*. *pumila* ‘Jinye’, whereas *U*. *pumila* ‘Jinye’ had a higher proportion of carotenoids, which may be the cause of the yellow leaves. Examination of the cell microstructure and RNA sequencing analysis showed that the leaf color and growth inhibition were mainly due to the following reasons: first, there were differences in the structure of the thylakoid grana layer. *U.* *pumila* L. has a normal chloroplast structure and clear thylakoid grana slice layer structure, with ordered and compact thylakoids. However, *U.* *pumila* ‘Jinye’ exhibited the grana lamella stacking failures and fewer thylakoid grana slice layers. As the pigment carrier and the key location for photosynthesis, the close stacking of thylakoid grana could combine more chlorophyll and promote efficient electron transfer promoting the photosynthesis reaction. In addition, *U.* *pumila* ‘Jinye’ had a lower capacity for light energy absorption, transformation, and transportation, carbon dioxide (CO_2_) fixation, lipopolysaccharide biosynthesis, auxin synthesis, and protein transport. The genes related to respiration and starch consumption were higher than those of *U.* *pumila* L., which indicated less energy accumulation caused the growth inhibition of *U*. *pumila* ‘Jinye’. Finally, compared with *U*. *pumila* ‘Jinye’, the transcription of genes related to stress resistance all showed an upward trend in *U.* *pumila* L. That is to say, *U.* *pumila* L. had a greater ability to resist adversity, which could maintain the stability of the intracellular environment and maintain normal progress of physiological metabolism. However, *U*. *pumila* ‘Jinye’ was more susceptible to changes in the external environment, which affected normal physiological metabolism. This study provides evidence for the main cause of growth inhibition in *U*. *pumila* ‘Jinye’, information for future cultivation, and information on the mutation mechanism for the breeding of colored leaf trees.

## 1. Introduction

Elm has a long history of use as a high-quality heavy hardwood famous for its resistance to drought, cold, and salt [1,2,3]. *Ulmus pumila* ‘Jinye’ is the natural mutant of *Ulmus pumila* L., which distributed across most of China. Due to its golden-yellow leaves and strong stress resistance, *U. pumila* ‘Jinye’ is widely used in landscaping in China. In addition, the foliage of *U. pumila* ‘Jinye’ contains many proteins and has good palatability, rendering it suitable for animal feed and feed processing. Moreover, its fruit and bark can be used in medicines [4,5,6].

In nature, the majority of colorful foliage plants are genetic mutants, and have great value in production and scientific research. Leaf color mutations are also known as chlorophyll-deficient; in leaf color mutations, mutant genes often directly or indirectly affect the synthesis or degradation of pigments to change pigment contents and proportions, which ultimately results in leaf color changes. At present, the molecular mechanism of leaf color mutation can be broadly classified into the following categories: (1) mutation of genes in the pathway of chlorophyll metabolism [7,8]; (2) mutation of chloroplast development related genes [9]; (3) mutation of genes in heme metabolism pathway [10]; (4) mutation of genes in carotenoid metabolism pathway [11]; (5) mutations in other genes encoding chloroplast proteins [12]. As the main pigment of photosynthesis, chlorophyll directly affects the photosynthetic efficiency of plants. Therefore, leaf coloration mutants are ideal specimen for photosynthesis research. However, the chlorophyll contents of leaf color mutants are lower than that of normal plants, which causes growth inhibition and even death. To date, numerous mutations have been found in *Zea mays* [13], *Pisum sativum* [14], *Nicotiana tabacum* [15], *Glycine max* [16], *Hordeum vulgare* [17], *Arabidopsis thaliana* [18], and *Oryza sativa* [19]. At present, *U. pumila* ‘Jinye’ is the most widely used new color variety in China, and its total industrial output value has exceeded 10 billion yuan (¥). Based on the importance of *U. pumila* ‘Jinye’ industry, we chose *U. Pumila* ‘Jinye’ as experimental material for research. In common with most leaf color mutants, *U. pumila* ‘Jinye’ exhibits growth inhibition. However, the mechanism of the mutation is unclear, and little is known about growth inhibition in of mutant plants, for example, whether any changes in the tissue structure of leaves can be observed, which normal physiological metabolic activities are affected, or whether the expression of growth-related genes are inhibited. In this study, we selected *U. pumila* L. and *U*. *pumila* ‘Jinye’ as specimens to investigate the growth inhibition mechanism at the physiological, cellular microstructural, and transcriptional levels, to provide information for future cultivation and the mutation for the breeding of colored leaf trees.

## 2. Results and Analysis

### 2.1. Growth and Physiological Factors Analysis

**Figure 1:**
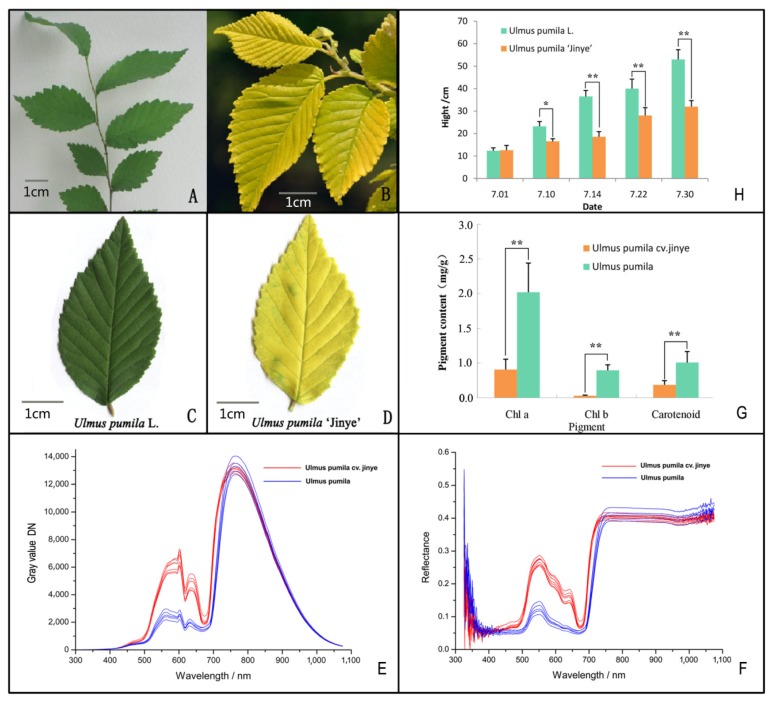
Phenotypic and physiological differences. (**A**) Branches of *Ulmus pumila* L.; (**B**) Branches of *U*. *pumila* ’Jinye’; (**C**) Leaves of *U*. *pumila* L.; (**D**) Leaves of *U*. *pumila* ‘Jinye’; (**E**) Gray value (digital number) of different elm leaves; (**F**) Spectral reflectance of different elm leaves; (**G**) Pigment content of different elm leaves; (**H**) Height growth of different elms. * mean significant difference (*p* < 0.05), ** mean very significant difference (*p* < 0.01).

As shown in Figure 1, there are significant differences in leaf color between *U*. *pumila* L. and *U*. *pumila* ‘Jinye’ (Figure 1A–D). Moreover, the reflectance spectrum result showed that the gray value of *U*. *pumila* ‘Jinye’ was higher than that of *U*. *pumila* L. within the visible wavelength range (400–800 nm). There were two distinct peaks (at 610 nm (yellow) and 640 nm (orange)) in the reflectance spectrum of *U*. *pumila* ‘Jinye’, whereas there were three peaks (at 560 nm (green), 610 nm (yellow), and 640 nm (orange)) in that of *U*. *pumila* L. (Figure 1E). The spectral reflectance also showed that *U*. *pumila* ‘Jinye’ had a high reflectivity at green and yellow wavelengths, whereas *U*. *pumila* L. only had high reflectivity in the green wavelength range (Figure 1F). The results indicated that *U. pumila* L. leaves can absorb large proportions of light energy for photosynthesis, whereas *U*. *pumila* ‘Jinye’ leaves dissipate most light energy in the form of reflection, and only a small part is absorbed for self-growth. The pigment contents of *U. pumila* L. were significantly higher than those of *U*. *pumila* ‘Jinye’. *U. pumila* L. had 3.75 times more chlorophyll a, 13.48 times more chlorophyll b, and 2.72 times more carotenoids than *U*. *pumila* ‘Jinye’ (Figure 1G). However, *U*. *pumila* ‘Jinye’ had a higher proportion of carotenoids, which explains the difference in coloration. The growth rate of *U. pumila* L. was significantly higher than that of *U*. *pumila* ‘Jinye’ (Figure 1H), indicating clear growth inhibition in *U*. *pumila* ‘Jinye’.

### 2.2. Photosynthetic Rate and Chlorophyll Fluorescence Analysis

**Figure 2:**
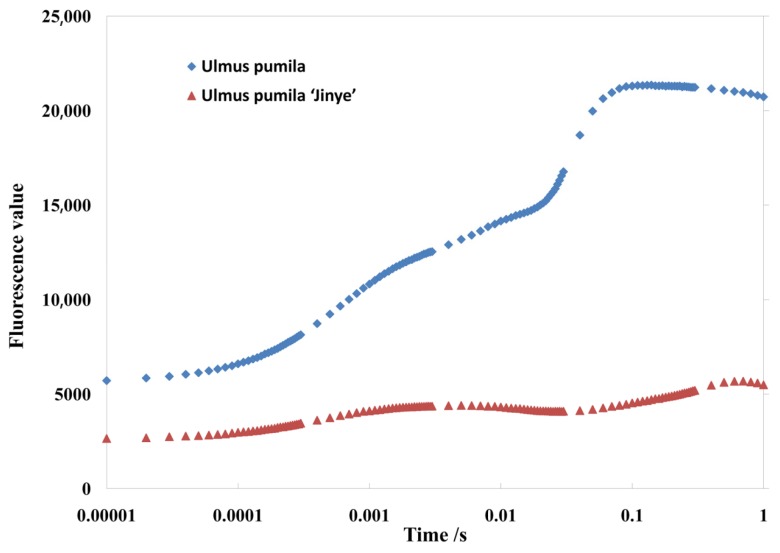
Fluorescence kinetics of O-J-I-P curve of different elms.

Photosynthesis is the basis of plant growth and development, and the rate of photosynthesis directly determines the growth rate of plants. Known from the analysis, there were significant differences in photosynthetic efficiency between *U. pumila* L. and *U*. *pumila* ‘Jinye’ (Table 1). The net photosynthetic rate (*Pn*) of *U. pumila* L. was significantly higher than that of *U*. *pumila* ‘Jinye’, which explains the growth inhibition of *U*. *pumila* ‘Jinye’. Moreover, the chlorophyll fluorescence kinetics curve of *U. pumila* L. was significantly higher than that of *U*. *pumila* ‘Jinye’ (Figure 2).

Following the JIP-test theory, the primary photochemical reactions of the photosystem II (PS II) reaction center were analyzed. *U*. *pumila* ‘Jinye’ had lower values of *Fo*, *Fm*, and *Fv*. The *S*m value of *U*. *pumila* ‘Jinye’ was significantly lower than that of *U. pumila* L., indicating that the plastoquinone (PQ) pool of the receptor side of the PS II reaction center was smaller, and less electrons entered the electron transport chain from the QA in *U*. *pumila* ’Jinye’. Moreover, the density of reaction centers (*RC/CS*_o_) was also smaller, showing damage to the PS II system. Moreover, the trapped (*TR_o_/CS_o_*), absorption (*ABS/CS*_o_) energy, and electron transport flux (*ET_o_/CS_o_*) of *U*. *pumila* ‘Jinye’ were significantly lower than those of *U. pumila* L. (Table 1). In other words, the ability to absorb light energy and the rate of electron transport in *U*. *pumila* ‘Jinye’ were lower, which limited the light reactions in photosynthesis. In addition, we also found that in terms of yield or flux ratio components, most energy was transferred to the light reaction center via electron transfer (a lower quantum ratio for heat dissipation) to promote the light reactions of photosynthesis (*φP_o_*, *ψ_O_*, *φE_o_*) of *U*. *pumila* L., which explains the higher photosynthetic efficiency of *U*. *pumila* L.

### 2.3. Cellular Microstructure Analysis

**Figure 3:**
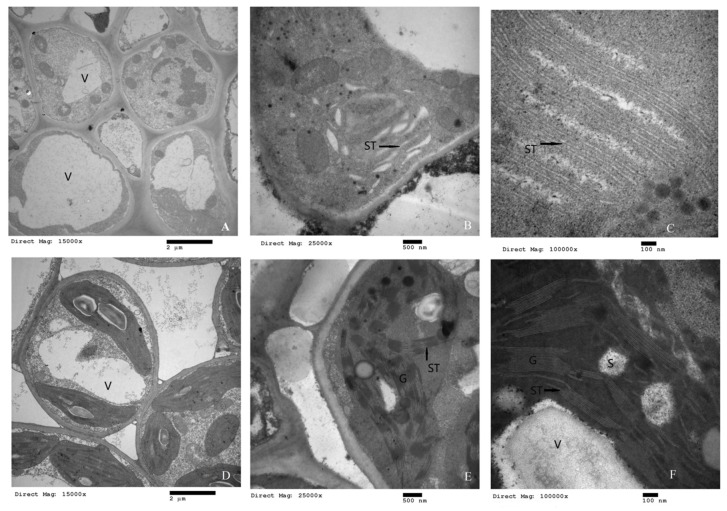
Comparison of chloroplast ultrastructures of (**A**–**C**) *Ulmus pumila* ’Jinye’ and (**D**–**F**) *Ulmus pumila* L. Note: (**A**–**C**) show the microstructure of chloroplasts with different magnification (15,000×, 25,000×, 100,000×) of *Ulmus pumila* ’Jinye’; (**D**–**F**) show the microstructure of chloroplasts with different magnification (15,000×, 25,000×, 100,000×) of *Ulmus pumila* L.; G—Granum; ST—Stoma thylakoid; S—Starch; V—Vacuole.

To investigate the reasons for the differences in pigment contents and growth inhibition, the microscopic structure of the chloroplasts was also analyzed in detail. Transmission electron microscopy results showed that there were significant differences in chloroplast structure between the two elms (Figure 3). There were significant differences in the shape and internal structure of mesophyll cells, chloroplasts, and the cell membrane system in *U*. *pumila* ‘Jinye’ compared to *U*. *pumila* L. The chloroplast development of *U*. *pumila* ‘Jinye’ was incomplete, and many mesophyll cells did not have chloroplasts (Figure 3A). The chloroplast membranes were thin (200 nm compared to 375 nm in *U*. *pumila* L.) with damaged edges, indicating imperfect development of the chloroplast membrane system in *U*. *pumila* ‘Jinye’ (Figure 3B). Compared with *U*. *pumila* L., the mesophyll cells were significantly smaller in *U*. *pumila* ‘Jinye’, including mesophyll cell length, width, and cell wall thickness (Table S1). The chloroplast volume of *U*. *pumila* ‘Jinye’ was also significantly smaller than that of *U*. *pumila* L. (Table S1). Moreover, the thylakoids of *U*. *pumila* ‘Jinye’ were swollen, and the stroma lamella and grana lamella of chloroplasts were distorted. Grana lamella stacking failure and fewer thylakoid grana slice layers (3–4 layers; Figure 3C) were observed in *U*. *pumila* ‘Jinye’, whereas close stacking of grana lamella and more thylakoid grana slice layers (24 layers) were observed in *U*. *pumila* L. (Figure 3D–F). Furthermore, *U*. *pumila* ‘Jinye’ had fewer total thylakoids, and fewer existed in the chloroplast stroma as stroma-thylakoids (ST). The number of grana thylakoids significantly increased in *U*. *pumila* L. (Table S1). Moreover, the stacking layers increased and a slight crosslinking structure between grana was observed. The grana lamellae were stacked tightly and formed a dense network structure between grana via ST crosslinking. As thylakoid membranes are the major reaction centers of photosynthesis, the state of grana stacking may directly affect the level of photosynthetic efficiency. In addition, thylakoids are the pigment carrier, including most of the photosynthetic pigments; therefore, the stacking state of thylakoid layers directly affects the accumulation of pigments. This is also probably the main reason for the differences in pigment contents and photosynthetic efficiency between *U. pumila* L. and *U*. *pumila* ‘Jinye’.

### 2.4. RNA Sequencing Analysis

After quality control, the sequencing primers, joints, and other low-quality reads were removed, and 12 Gb of clean data were obtained (Table 2). The guanine-cytosine (GC) contents of the two elms were similar (about 45.72%), and the Q30 bases of all of the samples exceeded 92%, which met the requirement for the follow-up test.

#### 2.4.1. DEG Analysis

**Figure 4:**
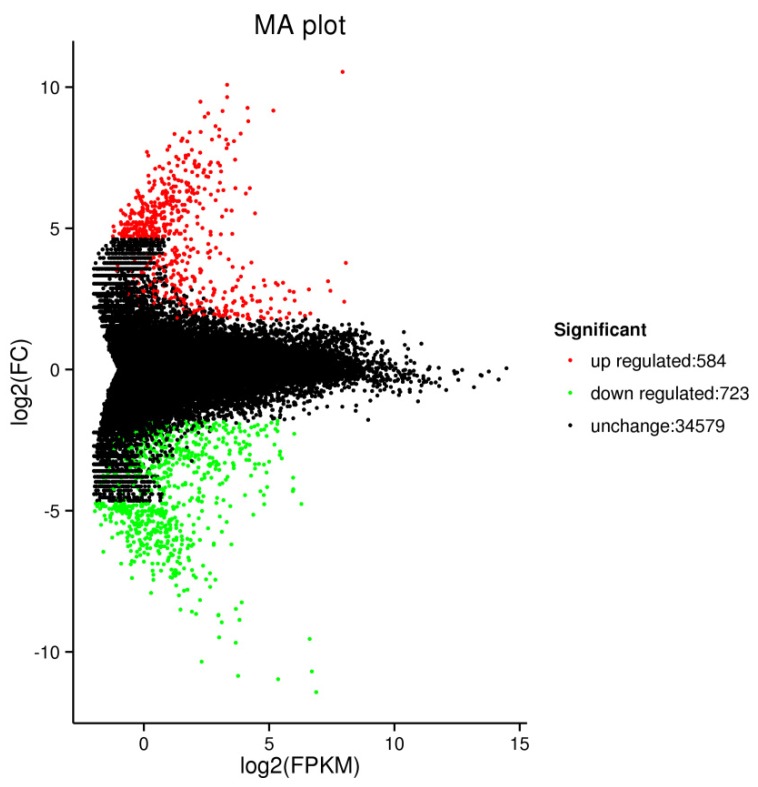
MA plot of differentially expressed genes (DEGs).

The MA plot showed that there were 1307 DEGs (Table S2.), of which 584 were up regulated and 723 were down regulated (Figure 4). It showed that the mutation of *U. pumila* ‘Jinye’ has great influence on gene expression. Based on the expression of genes in different samples, a total of 714 genes were annotated (including COG-193, GO-311, KEGG-401, KOG-494, Pfam, Swiss-Prot-460, and NR-688).

#### 2.4.2. COG Classification of DEGs

**Figure 5:**
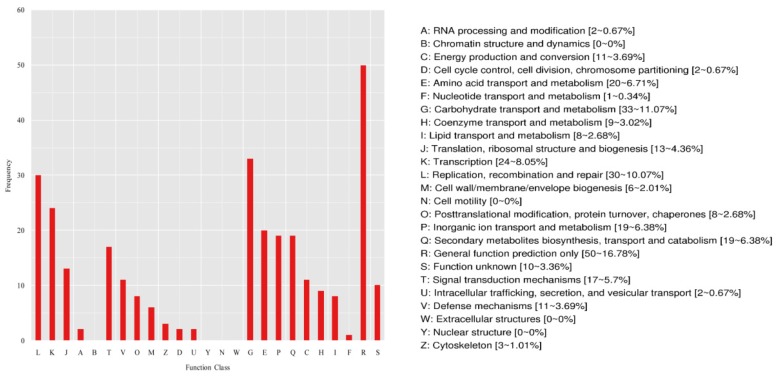
Clusters of orthologous group (COG) of proteins annotation classification of DEGs.

The COG database is constructed based on phylogenetic relationships among bacteria, algae, and eukaryotes, and enables immediate homological classification of gene products. Using COG classification, the DEGs were mainly concentrated in the following categories (Figure 5). In the information storage and processing, the DEGs were mainly involved in (L) replication, recombination, and repair (30, 10.07%), (K) transcription (24, 8.05%) and (J) translation, ribosomal structure and biogenesis (13, 4.36%); in the respect of cellular processes and signaling, the DEGs were mainly involved in (T) signal transduction mechanisms (17, 5.70%), (V) defense mechanisms (11, 3.69%) and (O) posttranslational modification, protein turnover, chaperones(8, 2.68%); in the metabolism part, the DEGs were mainly involved in (G) Carbohydrate transport and metabolism (33, 11.07%), (E) Amino acid transport and metabolism (20, 6.71%), (P) In organic ion transport and metabolism (19, 6.38%), (Q) Secondary metabolites biosynthesis, transport, and catabolism (19, 6.38%), and (C) Energy production and conversion (11, 3.69%).These results indicated that gene mutation of *U*. *pumila* ‘Jinye’ has a great impact on the expression of genes in carbohydrate accumulation, signal transduction mechanisms, stress tolerance, and amino acid transport.

#### 2.4.3. KOG Classification of DEGs

**Figure 6:**
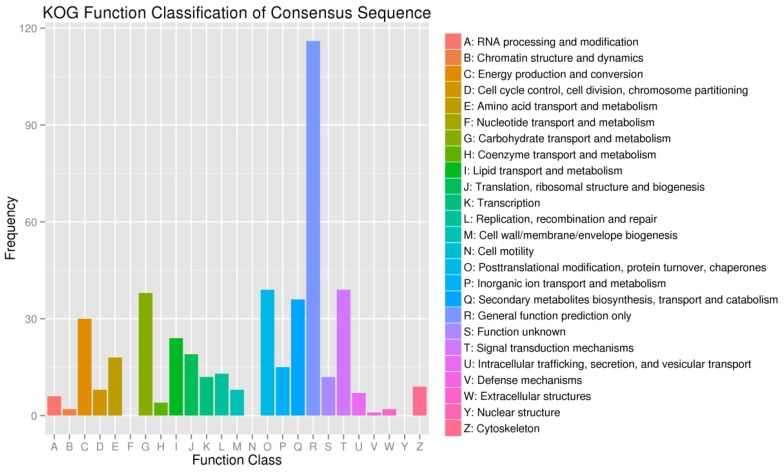
Eukaryotic orthologous group (KOG) annotation classification of DEGs.

Using KOG classification, the differentially expressed genes were mainly concentrated in the following categories (Figure 6): (R) general function prediction only (116, 25.33%), (T) signal transduction mechanisms (39, 8.52%), (O) posttranslational modification, protein turnover, and chaperones (39, 8.52%), (G) carbohydrate transport and metabolism (38, 8.30%), (Q) secondary metabolites biosynthesis, transport, and catabolism (36, 7.86%), and (C) energy production and conversion (30, 6.55%). There were significant differences in signal transduction mechanisms and energy transport, accumulation, and consumption between *U. pumila* and *U*. *pumila* ‘Jinye’. All above showed that gene mutation may have affected the physiological processes such as signal transduction and energy accumulation of *U*. *pumila* ‘Jinye’, and eventually inhibited the growth.

#### 2.4.4. KEGG Metabolism Pathway Analysis

**Figure 7:**
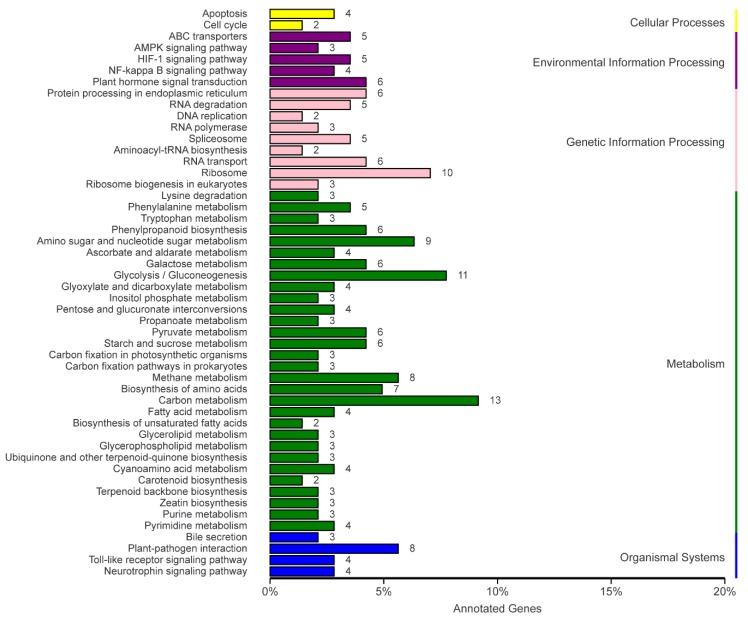
Kyoto Encyclopedia of Genes and Genomes (KEGG) classification of DEGs.

The KEGG database is the main public database for pathways. In organisms, different gene products are coordinated to perform biological functions, and pathway annotations of DEGs elucidate these functions. The KEGG pathway enrichment analysis showed that most DEGs were associated with metabolism (79) and genetic information processing (43), followed by environmental information processing (23), organismal systems (19), and cellular processes (6) (Figure 7). This indicated that the physiological metabolism levels of *U. Pumila* L. and *U*. *pumila* ‘Jinye’ were distinctly different.

According to the results of the KEGG database annotation, a total of 142 DEGs were annotated and aligned with 60 metabolic pathways (Table 3). Carbon metabolism contained the largest number of DEGs (13), followed by glycolysis/gluconeogenesis (11), ribosome (9), amino sugar and nucleotide sugar metabolism (10), methane metabolism (8), plant–pathogen interactions (8), biosynthesis of amino acids (7), protein processing in the endoplasmic reticulum (6), RNA transport (6), starch and sucrose metabolism (6), pyruvate metabolism (6), plant hormone signal transduction (6), phenylpropanoid biosynthesis (6), galactose metabolism (6), spliceosome (5), RNA degradation (5), HIF-1 signaling pathway (5), phenylalanine metabolism (5), and ABC transporters (5).

#### 2.4.5. DEG Pathway Analysis

**Figure 8:**
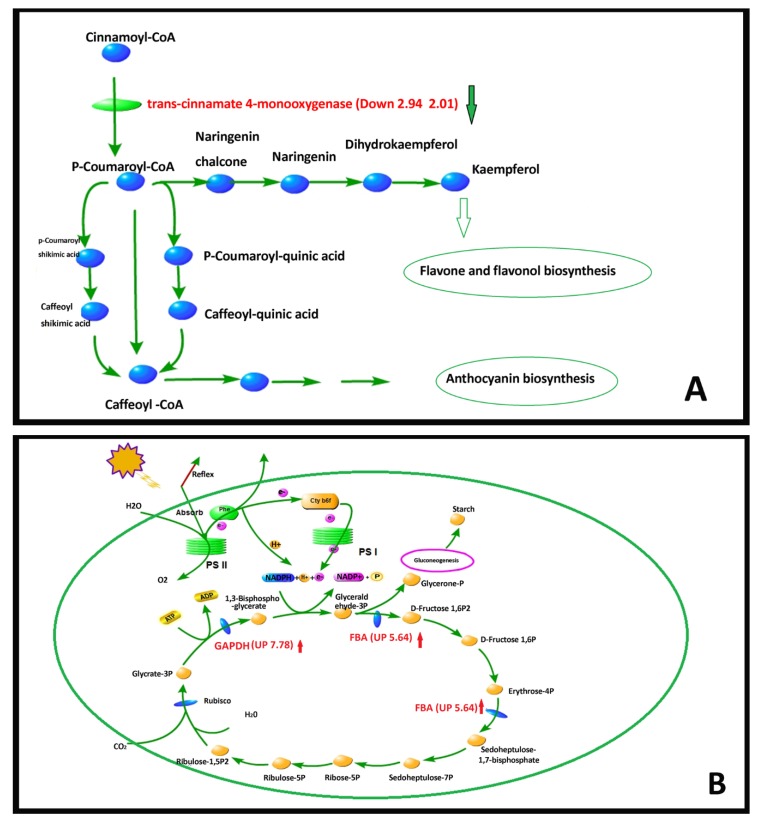

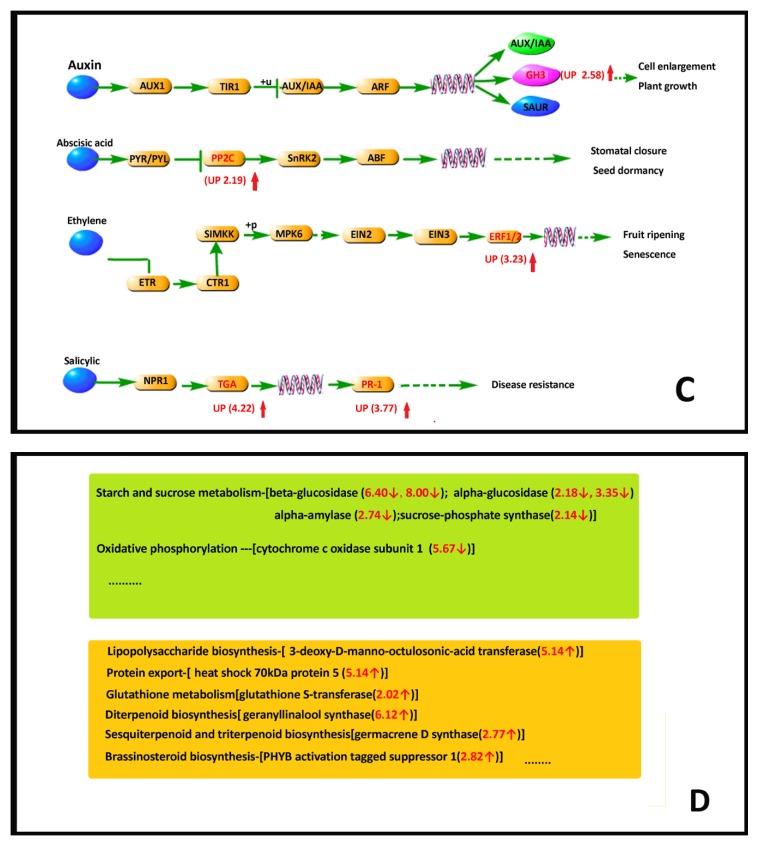
Pathway of differentially expressed genes. (**A**)The pathway of flavonoids biosynthesis; (**B**) The pathway of carbon fixation in photosynthetic organisms; (**C**) The pathway of plant hormone signal transduction; (**D**) The pathway of other DEGs pathway.

As we reported above, the carotenoid relative contents of *U*. *pumila* ‘Jinye’ were higher than those of *U. pumila* L., which explained the yellow color of the leaves. Combined with the KEGG database, we found that the carotenoid was down regulated in *U. pumila* L. Compared with *U*. *pumila* ‘Jinye’, the expression of genes related to abscisic acid 8”-hydroxylase [EC:1.14.13.93] and xanthoxin dehydrogenase [EC:1.1.1.288] was down regulated, which explains the high relative content of carotenoid in *U*. *pumila* ‘Jinye’ (Figure 8A). We also found that *U*. *pumila* ‘Jinye’ had a stronger capacity for flavonoid biosynthesis than *U*. *pumila* L. In the flavonoid biosynthesis pathway of *U. pumila* L., the genes associated with trans-cinnamate 4-monooxygenase enzyme [EC:1.14.13.11] were down regulated (Figure 8A). Trans-cinnamate 4-monooxygenase is the key rate-limiting enzyme in the regulation of Cinnamoyl-CoA to p-Cinnamoyl-CoA. The activity of the trans-cinnamate 4-monooxygenase enzyme can directly determine the efficiency of flavone and flavonol biosynthesis. The downregulation of the gene expression indicated that flavonoid biosynthesis in *U. pumila* L. was not vigorous, which also helps to explain the yellow leaves of *U*. *pumila* ‘Jinye’ from another perspective.

Photosynthesis is a biochemical process where green plants use organic carbon dioxide, water, and light energy to produce organic matter and release oxygen. It is the basis of plant growth and development and the main factor of productivity. The above analysis results showed that the chloroplast development of *U*. *pumila* ‘Jinye’ was incomplete, and many mesophyll cells did not have chloroplasts. Moreover, the ability to absorb light energy and the rate of electron transport in *U*. *pumila* ‘Jinye’ were lower, which limited the light reactions in photosynthesis. Photosynthesis is accomplished jointly by two interrelated stages of light reaction and dark reaction. The dark reaction also determines the rate of photosynthesis. CO_2_ assimilation is a very important process in photosynthesis. Additional analyses elucidated CO_2_ assimilation ability of *U*. *pumila* ‘Jinye’ is also lower than *U*. *pumila* L. (Figure 8B). In the carbon fixation pathway of *U. pumila*, the expression levels of phosphoenolpyruvate carboxykinase (ATP) [EC:4.1.1.49], glyceraldehyde 3-phosphate dehydrogenase (GADPH) [EC:1.2.1.12] and fructose-bisphosphate aldolase (class II) (FBA) [EC:4.1.2.13] were all up regulated. Under the catalysis of Rubisco (ribulose bisphosphate carboxylase/oxygenase) enzyme, RuBP is combined with CO_2_ to generate Glycrate-3P. Under the catalysis of GADPH enzyme, Glycrate-3P was turned into 1,3-Bisphospho-glycerafe and Glyceraldehyde-3P, by which completed the process of storing energy of photosynthesis. On the one hand, glyceraldehyde-3P can produce sucrose and starch through a series of reactions to promote the accumulation of organic matter; on the other hand, it can participate in the Calvin cycle under the action of FBA enzyme, and ultimately promote CO_2_ assimilation. The up-regulated expression of *GADPH* and *FBA* genes could enhance the CO_2_ immobilization capacity of plants and ultimately improve the photosynthesis rate of plants. That means *U. pumila* L. had stronger CO_2_ assimilation capacity than *U*. *pumila* ‘Jinye’. The photosynthesis of *U. pumila* ‘Jinye’ is inhibited, energy sources were less, organic matter accumulation rate was reduced, and plant growth was inhibited. This is the main reason for the growth inhibition of *U. pumila* ‘Jinye’.

Plant hormones are a class of organic substances produced by the plant’s own metabolism, which are closely related to the growth and development of plants. We also found that the expression of genes related to plant hormone signal transduction pathway were changed significantly in the two elms. Auxin can promote the growth of the plant by promoting the growth of the cells, especially the elongation of the cells. In this study, we found that the expression of GH3 (auxin responsive *GH3* gene family) was up-regulated in *U. pumila* L., which also explained the reason for the inhibition of the growth of *U. pumila* ‘Jinye’ from another point of view (Figure 8C). Abscisic acid, ethylene, and salicylic have many regulatory roles in response to biotic and abiotic stresses. In addition, the expression of genes related to abscisic acid, ethylene, and salicylic all showed an upward trend, which indicated that the resistance of *U. pumila* L. was stronger than *U. pumila* ‘Jinye’.

Through DEGs pathway analysis, we also found that *U. pumila* L. had a stronger ability to transport and accumulate organic matter than *U. pumila* ‘Jinye’. The genes related to lipopolysaccharide biosynthesis and protein transport were significantly higher in *U. pumila* L. than that in *U. pumila* ‘Jinye’. *U. pumila* L. has strong photosynthetic capacity and lower energy consumption. Moreover, *U. pumila* L. has less energy consumption. The genes related to the key respiration enzymes of starch and sucrose metabolism (beta-glucosidase, alpha-glucosidase, alpha-amylase, and sucrose-phosphate synthase) and oxidative phosphorylation (cytochrome c oxidase subunit 1) were all down regulated. This showed *U. pumila* L. has low energy consumption, while *U*. *pumila* ‘Jinye’ consumed more energy materials. Beyond that, the genes related to stress resistance in *U. pumila* L. were also up regulated, including those for glutathione transferase, diterpenoid biosynthesis, hydroperoxide dehydratase, brassinolide, ethylene, salicylic acid, terpenoid, cytochrome P450, and so on (Figure 8D and Figures S1–S3). This indicated that *U. pumila* L. has a stronger ability to maintain a stable cellular environment and protect routine functions during adverse conditions. By contrast, the stress resistance of *U*. *pumila* ‘Jinye’ was poor, as its physiology was disturbed easily by external factors.

#### 2.4.6. qPCR Verification Analysis

To further verify the accuracy of RNA sequencing (RNA seq), 13 genes (Table S3) were randomly selected from all of the genes for real-time Q-PCR validation (Figure S4). The results were consistent with those of RNA seq, indicating that the latter was reliable.

## 3. Materials and Methods

### 3.1. Materials

*U. pumila* L. and *U*. *pumila* ‘Jinye’ cuttings of identical growth and development were selected and planted in the experimental garden of the Agricultural University of Hebei (Baoding, China). There were no tall trees or buildings nearby to shade the experiment. During the growing season of July, the fully expanded functional leaves (the fourth leaves from the top) were collected and stored in liquid nitrogen prior to subsequent testing.

### 3.2. Determination of Physiological Factors

To reflect the differences in leaf colors accurately, the fourth leaves from the top branches were selected to measure the spectral reflectance by using an ASD Handheld2 (Analytical Spectral Devices, Boulder, CO, USA). Chlorophyll measurements for the various leaf samples were performed spectrophotometrically using an organic solvent extraction method [20] to compare pigment contents. Plant heights were measured once a week. All tests were repeated three times.

### 3.3. Photosynthetic Rate and Chlorophyll Fluorescence Analysis

The net photosynthetic rate of leaves was measured (three repetitions) using a LI-6400XT Portable Photosynthesis System (Li-COR Environmental, Lincoln, NE, USA) from 9:00 to 10:00 (a.m.). A Pocket PEA high-speed continuous excitation fluorescence spectrometer (British Bioscientific Co., Ltd., Norfolk, UK) was used to measured photosynthetic activity and efficiency (three repetitions). Based on the energy flow theory of biomembranes [21], Strasser [22] established a data analysis and processing method (JIP-test) for the rapid chlorophyll fluorescence induction curve. A number of other parameters can be obtained using the Strasser method. The minimal recorded fluorescence intensity *F*_o_ and the maximal recorded fluorescence intensity *F*_m_ can be used to calculate the variable fluorescence as *F*_v_ = *F*_m_ − *F*_o_, the relative variable fluorescence intensity at a given J-step is given by *V*_J_ = (*F*_J_ − *F*_o_)/(*F*_m_ − *F*_o_), and the normalized total complementary area above the O-J-I-P transit (reflecting single-turnover QA reduction events) is given by *S_m_* = (Area)/(*F*_m_ − *F*_o_). In addition, the following yield or flux ratio parameters can be calculated: the maximum quantum yield for primary photochemistry (at *t* = 0), *φP*_o_ = *TR*_o_/*ABS* = (1 − [*F*_o_/*F*_m_]); the probability that a trapped exciton moves an electron into the electron transport chain beyond QA (at *t* = 0), *ψ*o = *ET*_o_/*TR*_o_ = (1 − *V*_J_); the quantum yield for electron transport (at *t* = 0), *φE*_o_ = *ET*_o_/*ABS* = (1 − [*F*_o_/*F*_m_])*ψ*o; and the quantum ratio for thermal dissipation, *φD*o = 1 − *φPo*. Further parameters include those related to the phenomenological energy flux (per excited cross-section (CS)): the absorption flux per CS (at *t* = 0) is given by *ABS*/*CS*_o_ ≈ *F*_o_; the trapped energy flux per CS (at *t* = 0), *TR*_o_/*CS*_o_ = φ*P*_o_(*ABS*/*CS*_o_); the electron transport flux per CS (at *t* = 0), *ET*_o_/*CS*_o_ = *φE*_o_(*ABS*/*CS*_o_); and the dissipated energy flux per CS (at *t* = 0), *DI*_o_/*CS*_o_ = (*ABS*/*CS*_o_) − (*TR*_o_/*CS*_o_). Further parameters relate to the density of reaction centers, i.e., the density of RCs (QA reducing PS II reaction centers), *RC*/*CS*_o_ = *φP*_o_(*V*_J_/*M*_o_)(*ABS*/*CS*_o_), and to performance indices, i.e., the performance index on the absorption basis, *PI*_ABS_ = (*RC*/*ABS*)(*φP*_o_/[1 − *φP*_o_])(*ψ*_o_/[1 − *ψ*_o_]). A *t*-test was used for analysis with SPSS17 software.

### 3.4. Cellular Microstructure Analysis

Fully expanded functional leaves from the same site were selected and cut into 1 × 1 mm pieces, then fixed in 2.5% glutaraldehyde solution and stained with 3% uranyl acetate (*w*/*v*) and lead citrate. Sections were examined and photographed with a JEM-2000EX transmission electron microscope with an acceleration voltage of 80 kV to explore the differences in chloroplast structure at the cellular and subcellular levels. The microstructures of chloroplasts with different magnification were observed (randomly selecting five visual fields). The microstructural differences of different elm trees were compared by *t*-test with SPSS17 software.

### 3.5. RNA Extraction, Library Construction, and Sequencing

Total RNA was extracted using an EASYExPLUS Plant RNA Kit (Cyrus, Beijing, China). A Qubit 2.0 Fluorometer (Life Technologies, Carlsbad, CA, USA) and an Agilent 2100 bioanalyzer (Agilent Technologies, Palo Alto, Santa Clara, CA, USA) were used to detect the purity, concentration, and integrity of the RNA samples. The library was constructed from the samples and quantified using quantitative polymerase chain reaction (qPCR) to ensure quality. Briefly, eukaryotic mRNA was concentrated using magnetic beads with Oligo (dT). The mRNA was interrupted randomly with Fragment Buffer. First-strand cDNA was synthesized with mRNA as a template using hexanucleotide random hexamers, after which the second-strand cDNA was synthesized using a buffer, dNTPs, RNase H, and DNA polymerase I. Then, AMPure XP beads were used to purify the cDNA. The purified bi-strand cDNA was used for end repair, to add the tail, and connect the sequencing connection. Then, AMPure XP beads were used to select the fragment size. The cDNA library was enriched using PCR. Finally, the library was constructed using the Qubit 2.0 fluorometer to detect concentrations and the Agilent 2100 was used to detect the inserted fragment sizes. The library was quantified using qPCR to ensure the quality. Then HiSeq 2500 was used for high-throughput sequencing.

### 3.6. Illumina Sequencing, Assembly, and Functional Annotation

After the raw data were obtained, the adapter, primer sequences, and low-quality data were removed to acquire high-quality reads. Trinity software was used to assemble the sequencing data, then compared with the non-redundant (Nr) Swiss-Prot, Gene Ontology (GO), clusters of orthologous groups (COGs) of proteins, eukaryotic orthologous groups (KOG), and the Kyoto Encyclopedia of Genes and Genomes (KEGG) databases to obtain annotation information [23,24,25,26,27,28,29,30]. The Benjamini–Hochberg approach was used to correct significant *p*-values obtained from previous hypothesis tests in the differential gene screening process. The false discovery rate (FDR) < 0.01 and a fold change (FC) > 2 were used as standards [31] to screen for differentially expressed genes (DEGs). Then the DEGs were subjected to COG, KOG, GO, and KEGG pathway enrichment analysis.

### 3.7. Real Time Q-PCR Verification

Total RNA was extracted using an EASYExPLUS Plant RNA Kit (Cyrus, Beijing, China). Reverse transcription of the first cDNA chain was performed with a reverse transcription kit (Kangwei, Beijing, China). cDNA synthesized by reverse transcription was utilized as the template, and 2× SYBR Green qPCR Mix was used for fluorescence quantitative PCR (three technical and three biological repeats). The reaction system (20 μL) consisted of 10 μL 2× SYBR qPCR Mix, 0.5 μL forward primer (10 μM), 0.5 μL reverse primer (10 μM), 1 μL template, and 8 μL ddH_2_O. The reaction procedure was as follows: pre-degeneration at 95 °C for 5 min, denaturation at 95 °C for 10 s, and renaturation at 55 °C for 30 s. Fluorescence quantitative PCR primers were designed according to the sequence information of the target genes using Primer Premier 5.

## 4. Discussion and Conclusions

Nearly every color imaginable has been observed in the foliage of plants. The colors of plant leaves are directly affected by pigment content and proportion in plant cells [32,33]. There are considerable differences in color among different pigments. Chlorophyll, as the main pigment of photosynthesis, make leaves green; anthocyanin make leaves red; and carotenoids and flavonoids make leaves yellow. Due to the superposition of color, different pigment ratios in flowers, fruits, and leaves result in different colors. Mutant genes often directly or indirectly affect the synthesis or degradation of pigments, change the pigment contents and proportions, and ultimately change the color of leaves [34,35]. The chlorophyll contents in the vast majority of mutants are low, which affects photosynthetic efficiency, production, and even death. In our study, the pigment contents and compositions of the two elms were significantly different. *U. pumila* L. had higher pigments contents (chlorophyll a, chlorophyll b, and carotenoids) than *U. pumila* ‘Jinye’, whereas the relative content of carotenoids was higher in *U. pumila* ‘Jinye’. Moreover, RNA seq results also showed that the genes associated with carotenoid and flavonoid biosynthesis were all were up regulated in *U. pumila* ‘Jinye’. These may be the main reasons for the golden color of *U*. *pumila* ‘Jinye’ leaves.

Thylakoids are the pigment carrier, and the stacking state of thylakoid layers directly affects the accumulation of pigments. Numerous studies have shown that although leaf color mutation mechanisms differ, the chloroplast structure of most mutant plants is imperfect [36,37]. In this study, the shape and internal structure of mesophyll cells, chloroplasts, and the cell membrane system were significantly different in *U*. *pumila* ‘Jinye’. The mesophyll cells and chloroplasts were smaller and rounder, the thylakoids were swollen, and the stroma lamella and grana lamella of chloroplasts were distorted in *U*. *pumila* ‘Jinye’. In contrast, *U*. *pumila* L. had a normal chloroplast structure, with a more obvious thylakoid grana slice layer structure and well-ordered and compact thylakoids. As the carrier of photosynthetic pigments and the major site of photosynthesis, the stacking state of thylakoid layers directly affects the pigment content and photosynthetic efficiency, which may be the main reason for the growth inhibition of *U*. *pumila* ‘Jinye’. Compact thylakoid arrangements promote photosynthesis by facilitating electron transfer, which drives the light reactions of photosynthesis.

Photosynthesis is a critical pathway for synthesizing organic compounds that provide the energy sources for plant growth and development. Many studies have indicated that there is a close relationship between stress resistance and respiration and photosynthesis in plants. Stress may influence photosynthetic electron transfer and thylakoid structure, affect photosynthesis and carbon assimilation, and ultimately affect the photosynthetic rate. Plants with a higher photosynthetic capacity tend to have higher stress resistance. We introduced a source and sink theory to explain the growth inhibition of *U*. *pumila* ‘Jinye’ more clearly. Compared with *U. pumila* L., there are few energy sources and greater energy consumption, leading to growth inhibition in *U*. *Pumila* ‘Jinye’.

In this study, *U. pumila* L. had stable and efficient electron transfer, CO_2_ fixation capacity, lipopolysaccharide biosynthesis, auxin synthesis, and protein transport, as well as lower energy consumption. Compared to *U*. *pumila* ‘Jinye’, it had more stable and effective photosynthetic systems and material transportation. In addition, the plant itself is stronger and has a greater ability to resist stress. In contrast, due to the damage to photosynthetic structures caused by gene mutations of *U*. *pumila* ‘Jinye’, photosynthetic efficiency in this plant was reduced greatly, which caused clear problems for energy and growth. Furthermore, metabolism was reduced and resistance to environmental stresses was compromised. This was the main driver for the upregulation of stress-related genes such as glutathione transferase, hydroperoxide dehydratase, brassinolide, ethylene, salicylic acid, terpenoids, and cytochrome P450. Therefore, *U. pumila* L. was well equipped to maintain a stable intracellular environment and resist stress, whereas the physiology of *U*. *pumila* ‘Jinye’ was easily disturbed by external stressors. For example, *U*. *pumila* ‘Jinye’ is more vulnerable to diseases and insect pests, and its leaves appear albino, withered, or even dead in high-temperature or high-light intensity environments (August). Zhang [4] found another interesting phenomenon in which the leaf color will gradually change from yellow to green in shaded conditions. Moreover, growth was not inhibited significantly under shaded conditions. This is the first systematic study of growth differences between *U*. *pumila* ‘Jinye’ and *U. pumila* L. In this study, we preliminarily identified the reason for the growth inhibition of *U*. *pumila* ‘Jinye’, which could provide information for further gene mapping and other follow-up research. However, there are still many unresolved issues, such as stacked grana lamellae and material transport. In addition, the underlying mechanisms remain unclear and require further study.

## Figures and Tables

**Table 1:** Photosynthetic and chlorophyll fluorescence parameters.

Formulae and Terms		*U. pumila* L.	*U. pumila* ’Jinye’
Net photosynthetic rate	*Pn*	17.41 ± 1.42 ^a^	12.57 ± 2.80 ^b^
Fluorescence intensity	*Fo*	5695.32 ± 468.22 ^a^	2657.62 ± 248.74 ^b^
	*Fm*	21,337.45 ± 715.39 ^a^	5686.73 ± 482.54 ^b^
	*Fv*	15,642.73 ± 657.22 ^a^	3029.86 ± 397.61 ^b^
Yields or flux ratios	*φPo*	0.73 ± 0.13 ^a^	0.53 ± 0.08 ^b^
	*ψO*	0.84 ± 0.11 ^a^	0.73 ± 0.11 ^b^
	*φEo*	0.62 ± 0.07 ^a^	0.39 ± 0.04 ^b^
	*φDo*	0.27 ± 0.04 ^b^	0.47 ± 0.05 ^a^
Phenomenological energy fluxes	*ABS/CS_O_*	5695.73 ± 451.35 ^a^	2657.06 ± 426.33 ^b^
	*TR_O_/CS_O_*	4174.96 ± 391.57 ^a^	1415.42 ± 206.54 ^b^
	*ET_O_/CS_O_*	3523.71 ± 299.36 ^a^	1039.25 ± 255.32 ^b^
	*DI_O_/CS_O_*	1520.04 ± 268.13 ^a^	1241.58 ± 143.68 ^b^
Density of reaction centers	*RC/CS_O_*	2823.42 ± 162.47 ^a^	890.16 ± 118.67 ^b^

The results were expressed as average ± SD. *Pn*: Net photosynthetic rate; *Fo*: Minimal recorded fluorescence intensity; *Fm*: Maximal recorded fluorescence intensity; *φPo*: Maximum quantum yield for primary photochemistry (at *t* = 0); *ψo*: Probability that a trapped exciton moves an electron into the electron transport chain beyond QA (at *t* = 0); *φEo*: Quantum yield for electron transport (at *t* = 0); *φDo*: Quantum ratio for heat dissipation; *ABS/CSo*: Absorption flux per *CS* (at *t* = 0); *TRo/CSo*: Trapped energy flux per *CS* (at *t* = 0); *ETo/CSo*: Electron transport flux per *CS* (at *t* = 0); *DIo/CSo*: Dissipated energy flux per *CS* (at *t* = 0); *RC/CSo*: Density of *RCs* (QA-reducing PS II reaction centers). Different letter representations significant difference between different elms (*p* < 0.05).

**Table 2:** Sequencing data evaluation statistics.

Samples	Read Number	Base Number	GC Content	% ≥ Q30	Mapped Reads	Mapped Ratio
*U. pumila* L.	25,920,122	7,815,753,169	45.72%	92.08%	21,715,012	83.78%
*U. pumila* ‘Jinye’	26,788,118	8,080,109,712	45.70%	92.40%	22,450,089	83.81%

**Table 3:** Differentially expressed unigenes with significantly enriched pathways.

KEGG Pathway	DEG	Total	Enrichment Factor	*p*-Value	Corrected *p*-Value
Carbon metabolism	13	643	1.22	0.27	1.00
Glycolysis/Gluconeogenesis	11	315	2.11	0.02	1.00
Ribosome	10	823	0.73	0.89	1.00
Amino sugar and nucleotide sugar metabolism	9	192	2.83	0.00	0.30
Methane metabolism	8	178	2.71	0.01	0.63
Plant–pathogen interaction	8	239	2.02	0.04	1.00
Biosynthesis of amino acids	7	480	0.88	0.69	1.00
Galactose metabolism	6	114	3.18	0.01	0.77
Starch and sucrose metabolism	6	265	1.37	0.28	1.00
Pyruvate metabolism	6	254	1.43	0.24	1.00
Phenylpropanoid biosynthesis	6	171	2.12	0.06	1.00
RNA transport	6	317	1.14	0.43	1.00
Plant hormone signal transduction	6	189	1.92	0.09	1.00
Protein processing in endoplasmic reticulum	6	402	0.90	0.66	1.00
Phenylalanine metabolism	5	116	2.60	0.04	1.00
ABC transporters	5	56	5.39	0.00	0.15
RNA degradation	5	208	1.45	0.26	1.00
Spliceosome	5	350	0.86	0.69	1.00
HIF-1 signaling pathway	5	150	2.01	0.10	1.00
Pentose and glucuronate interconversions	4	140	1.73	0.20	1.00
Ascorbate and aldarate metabolism	4	90	2.68	0.06	1.00
Pyrimidine metabolism	4	221	1.09	0.50	1.00
Cyanoamino acid metabolism	4	67	3.61	0.02	1.00
Glyoxylate and dicarboxylate metabolism	4	187	1.29	0.37	1.00
Fatty acid metabolism	4	178	1.36	0.34	1.00
NF-kappa B signaling pathway	4	79	3.06	0.04	1.00
Apoptosis	4	110	2.20	0.11	1.00
Toll-like receptor signaling pathway	4	111	2.18	0.11	1.00
Neurotrophin signaling pathway	4	165	1.46	0.29	1.00
Ubiquinone and other terpenoid-quinone biosynthesis	3	48	3.78	0.04	1.00
Purine metabolism	3	300	0.60	0.88	1.00
Lysine degradation	3	98	1.85	0.22	1.00
Tryptophan metabolism	3	99	1.83	0.23	1.00
Glycerolipid metabolism	3	116	1.56	0.30	1.00
Inositol phosphate metabolism	3	103	1.76	0.24	1.00
Glycerophospholipid metabolism	3	143	1.27	0.42	1.00
Propanoate metabolism	3	98	1.85	0.22	1.00
Carbon fixation in photosynthetic organisms	3	163	1.11	0.51	1.00
Carbon fixation pathways in prokaryotes	3	110	1.65	0.27	1.00
Terpenoid backbone biosynthesis	3	75	2.42	0.13	1.00
Zeatin biosynthesis	3	23	7.88	0.01	0.41
Ribosome biogenesis in eukaryotes	3	183	0.99	0.59	1.00
RNA polymerase	3	74	2.45	0.12	1.00
AMPK signaling pathway	3	213	0.85	0.69	1.00
Bile secretion	3	64	2.83	0.09	1.00
Fructose and mannose metabolism	2	109	1.11	0.54	1.00
Fatty acid degradation	2	129	0.94	0.63	1.00
Photosynthesis	2	56	2.16	0.24	1.00
Valine, leucine, and isoleucine degradation	2	128	0.94	0.63	1.00
Glutathione metabolism	2	133	0.91	0.65	1.00
Riboflavin metabolism	2	24	5.03	0.06	1.00
Carotenoid biosynthesis	2	29	4.17	0.08	1.00
Nitrogen metabolism	2	43	2.81	0.16	1.00
Flavonoid biosynthesis	2	38	3.18	0.13	1.00
Stilbenoid, diarylheptanoid, and gingerol biosynthesis	2	24	5.03	0.06	1.00
Tropane, piperidine, and pyridine alkaloid biosynthesis	2	46	2.63	0.18	1.00
Aminoacyl-tRNA biosynthesis	2	123	0.98	0.61	1.00
Biosynthesis of unsaturated fatty acids	2	84	1.44	0.41	1.00
Degradation of aromatic compounds	2	23	5.25	0.05	1.00
DNA replication	2	92	1.31	0.45	1.00
Protein export	2	78	1.55	0.37	1.00
Cell cycle	2	228	0.53	0.90	1.00
Ubiquitin mediated proteolysis	2	220	0.55	0.88	1.00
Endocytosis	2	211	0.57	0.87	1.00
Insulin signaling pathway	2	220	0.55	0.88	1.00
Estrogen signaling pathway	2	125	0.97	0.62	1.00
Thyroid hormone synthesis	2	56	2.16	0.24	1.00

## Supplementary Materials

Supplementary materials can be found at https://www.mdpi.com/1422-0067/20/17/4227/s1.

## References

[1] Airy Show H.K., Willis’s J.C. (1973). A Dictionary of the Flowering Plant and Ferns.

[2] Dias M.C., Pinto G., Santos C. (2011). Acclimatization of micropropagated plantlets induces an antioxidative burst: A case study with *Ulmus minor* Mill. Photosynthetica.

[3] Dias M.C., Oliveira H., Costa A., Santos C. (2014). Improving elms performance under drought stress: The pretreatment with abscisic acid. Environ. Exp. Bot..

[4] Zhang S., Zuo L.H., Zhang J., Chen P.F., Wang J.M., Yang M.S. (2017). Transcriptome analysis of *Ulmus pumila* ‘Jinye’ responses to different shading involved in chlorophyll metabolism. Tree Genet. Genomes.

[5] Zuo L.H., Shang A.Q., Zhang S., Yu X.Y., Ren Y.C., Yang M.S., Wang J.M. (2017). The first complete chloroplast genome sequences of *Ulmus* species by de novo sequencing: Genome comparative and taxonomic position analysis. PLoS ONE.

[6] Dias M.C., Pinto G., Guerra C., Jesus C., Amaral J., Santos C. (2013). Effect of irradiance during acclimatization on content of proline and phytohormones in micropropagated *Ulmus minor*. Biol. Plant.

[7] Pontier D., Albrieux C., Joyard J., Lagrange T., Block M.A. (2007). Knock-out of the magnesium protoporphyrin IX methyltransferase gene in *Arabidopsis*. Effects on chloroplast development and on chloroplast-to-nucleus signaling. J. Biol. Chem..

[8] Kim Y.K., Lee J.Y., Cho H.S., Lee S.S., Ha H.J., Kim S., Choi D., Pai H.S. (2005). Inactivation of organellar glutamyl-and seryl-tRNA synthetases leads to developmental arrest of chloroplasts and mitochondria in higher plants. J. Biol. Chem..

[9] Wang P.R., Gao J.X., Wan C.M., Zhang F.T., Xu Z.J., Huang X.Q., Sun X.Q., Deng X.J. (2010). Divinyl chlorophyll(ide) a can be converted to monovinyl chlorophyll(ide) a by a divinyl reductase in rice. Plant Physiol..

[10] Patrick G.S., Matthew J.T. (2008). Light signalling pathways regulating the Mg-chelatase branchpoint of chlorophyll synthesis during de-etiolation in Arabidopsis thaliana. Photochem. Photobiol. Sci..

[11] Terry M.J., Kendriek R.E. (1999). Feedbaek inhibition of chlorophyll synthesis in the phytochrome chromophore deficient aurea and yellow-green-2 mutants of tomato. Plant Physiol..

[12] Gray J.C. (2003). Chloroplast-to-nucleus signalling: A role for Mg-protoporphyrin. Trends Genet..

[13] Lonosky P.M., Zhang X.S., Honavar V.G. (2004). A proteomic analysis of maize chloroplast biogenesis. Plant Physiol..

[14] Highkin H., Boardman N.K., Goodchild D.J. (1969). Photosynthetic studies on a pea mutant deficient in chlorophyll. Plant Physiol..

[15] Okabe K., Schmed G.H., Straub J. (1977). Genetic characterization and high efficiency photosynthesis of an aurea mutant of tobacco. Plant Physiol..

[16] Stockinger E.J., Walling L.L. (1994). A chlorophyll a/b-binding protein gene from soybean (*Glycine max* [L.] Merr.). Plant Physiol..

[17] Preiss S., Thornber J.P. (1995). Stability of the apoproteins of light-harvesting complex and during biogenesis of thylakoids in the chlorophyll b-less barley mutant chlorian f2. Plant Physiol..

[18] Carol P., Bisanz C., Breitenbach J., Sandmann G., Mache R., Coupland G., Kuntz M. (1999). Mutations in the Arabidopsis gene IMMUTANTS cause a variegated phenotype by inactivating a chloroplast terminal oxidase associated with phytoene desaturation. Plant Cell.

[19] Jung K.H., Hur J., Ryu C.H., Choi Y.J., Chung Y.Y., Miyao A.Y., Hirochika H., An G. (2003). Characterization of a chlorophyll-deficient mutant using the T-DNA gene-trap system. Plant Cell Physiol..

[20] Zou Q. (2000). Experimental Instruction of Plant Physiology.

[21] Strasser R.J., Akoyunoglou G. (1981). The Grouping Model of Plant Photosynthesis: Heterogeneity of Photosynthetic Units in Thylakoids. Photosynthesis III. Structure and Molecular Organization of the Photosynthetic Apparatus.

[22] Strasser R.J., Tsimill-Michael M., Srivastava A., Govindjee P.G. (2004). Analysis of the chlorophyll a fluorescence transient. Advances in Photosynthesis and Respiration.

[23] Eddy S.R. (1998). Profile hidden Markov models. Bioinformatics.

[24] Ashburner M., Ball C.A., Blake J.A., Botstein D., Butler H., Cherry J.M., Davis A.P., Dolinski K., Dwight S.S., Eppig J.T. (2000). Gene ontology: Tool for the unification of biology. Nat. Genet..

[25] Tatusov R.L., Galperin M.Y., Natale D.A. (2000). The COG database: A tool for genome scale analysis of protein functions and evolution. Nucleic Acids Res..

[26] Apweiler R., Bairoch A., Wu C.H., Barker W.C., Noeckmann B., Ferro S., Gasteiger E., Huang H.Z., Lopez R., Magrane M. (2004). UniProt: The Universal Protein knowledgebase. Nucleic Acids Res..

[27] Kanehisa M., Goto S., Kawashima S., Okuno Y., Hattori M. (2004). The KEGG resource for deciphering the genome. Nucleic Acids Res..

[28] Koonin E.V., Fedorova N.D., Jackson J.D., Jacobs A.R., Krylov D.M., Makarova K.S., Mazumder R., Mekhedov S.L., Nikolskaya A.N., Rao B.S. (2004). A comprehensive evolutionary classification of proteins encoded in complete eukaryotic genomes. Genome Biol..

[29] Deng Y.Y., Li J.Q., Wu S.F., Zhu Y.P., Chen Y.W., He F.C. (2006). Integrated nr database in protein annotation system and its localization. Comput. Eng..

[30] Xie C., Mao X., Huang J., Ding Y., Wu J., Dong S., Kong L., Gao G., Li C.Y., Wei L.P. (2011). KOBAS 2.0: A web server for annotation and identification of enriched pathways and diseases. Nucleic Acids Res..

[31] Leng N., Dawson J.A., Thomson J.A., Ruotti V., Rissman A.I., Smits B.M.G., Haag J.D., Gould M.N., Ron M., Kendziorski C. (2013). EBSeq: An empirical bayes hierarchical model for inference in RNA-seq experiments. Bioinformatics.

[32] Close D.C., Beadle C.L. (2002). The ecophysiology of foliar anthocyanin. Bot. Rev..

[33] Hughes N.M., Carpenter K.L., Cannon J.G. (2014). Estimating contribution of anthocyanin pigments to osmotic adjustment during winter leaf reddening. J. Plant Physiol..

[34] Lin C.S., Lai Y.H., Sun C.W., Liu N.T., Tsay H.S., Chang W.C., Teremy J., Chen W. (2006). Identification of ESTs differentially expressed in green and albino mutantbamboo (Bambusaedulis) by suppressive subtractive hybridization (SSH) and microarray analysis. Plant Cell Tissue Organ. Cult..

[35] Hörtensteiner S. (2009). Stay-green regulates chlorophyll and chlorophyll-binding protein degradation during senescence. Trends Plant Sci..

[36] Melis A., Yocum C.F., Heichel I.F. (1996). Excitation energy transfer: Functional and dynamic aspects of Lhc(cab) proteins. Oxygenic Photosynthesis: The Light Reactions.

[37] Yang Y.L., Xu J., Rao Y.C., Zeng Y.J., Liu H.J., Zheng T.T., Zhang G.H., Hu J., Guo L.B., Qian Q. (2016). Cloning and functional analysis of pale-green leaf(PGL10) in rice (*Oryza sativa* L.). Plant Growth Regul..

