# Supplementary material for: The Reason for Growth Inhibition of Ulmus pumila ‘Jinye’: Lower Resistance and Abnormal Development of Chloroplasts Slow Down the Accumulation of Energy
Source: Int J Mol Sci. 2019 Aug 29;20(17):4227. doi: 10.3390/ijms20174227 (PMC6747506; doi:10.3390/ijms20174227)
Supplement: Supplementary file 1 [file ijms-20-04227-s001.zip › ijms-559609-supplementary files/S1 table.docx]

**Table S1.** Comparative analysis of cell microstructure.

| Microstructural parameters | *U*. *pumila* L. | *U*. *pumila* ‘Jinye’ |
| --- | --- | --- |
| Mesophyll cell length (um) | 7.42±1.35 ^a^ | 4.33±0.95 ^b^ |
| Mesophyll cell width (um) | 6.08±1.54 ^a^ | 4.25±1.62 ^b^ |
| Mesophyll cell wall thickness (um) | 0.27±0.03 ^a^ | 0.09±0.02 ^b^ |
| Chloroplast length (um) | 5.42±1.47 ^a^ | 0.92±0.08 ^b^ |
| Chloroplast width (um) | 1.50±0.43 ^a^ | 0.46±0.06 ^b^ |
| Chloroplast wall thickness (nm) | 375.00±25.68 ^a^ | 200.00±15.28 ^b^ |
| thylakoid grana slice layers | 23.62±5.69 ^a^ | 3.57±1.28 ^b^ |
| thylakoid grana thickness (nm) | 8.20±2.55 ^b^ | 10.16±3.56 ^a^ |
| cross-link | 4.22±2.33 ^a^ | 0.00±0 ^b^ |

Note: Different letter representations significant difference between different elms (p < 0.05).

Table S2 The detail of differentially expressed genes (DEGs)

Table S3 The primers information for qPCR

Figure S1 Pathway of lipopolysaccharide biosynthesis

Figure S2 Pathway of diterpenoid biosynthesis

Figure S3 Pathway of sesquiterpenoid and triterpenoid biosynthesis

Figure S4. Real-time qPCR analysis of 13 randomly selected genes. Note: The ordinate is the relative expression (log2 [T/CK]). 1–13 denote the relative expression of unigenes 27871, 32570, 36564, 34113, 7215, 19841, 7389, 18912, 3918, 21684, 7436, 39536, and 33702, respectively. Real-time Q-PCR was performed for three independent biological replicates, each containing three technical replicates.
