# Supplementary figures and images for: The Reason for Growth Inhibition of Ulmus pumila ‘Jinye’: Lower Resistance and Abnormal Development of Chloroplasts Slow Down the Accumulation of Energy
Source: Int J Mol Sci. 2019 Aug 29;20(17):4227. doi: 10.3390/ijms20174227 (PMC6747506; doi:10.3390/ijms20174227)

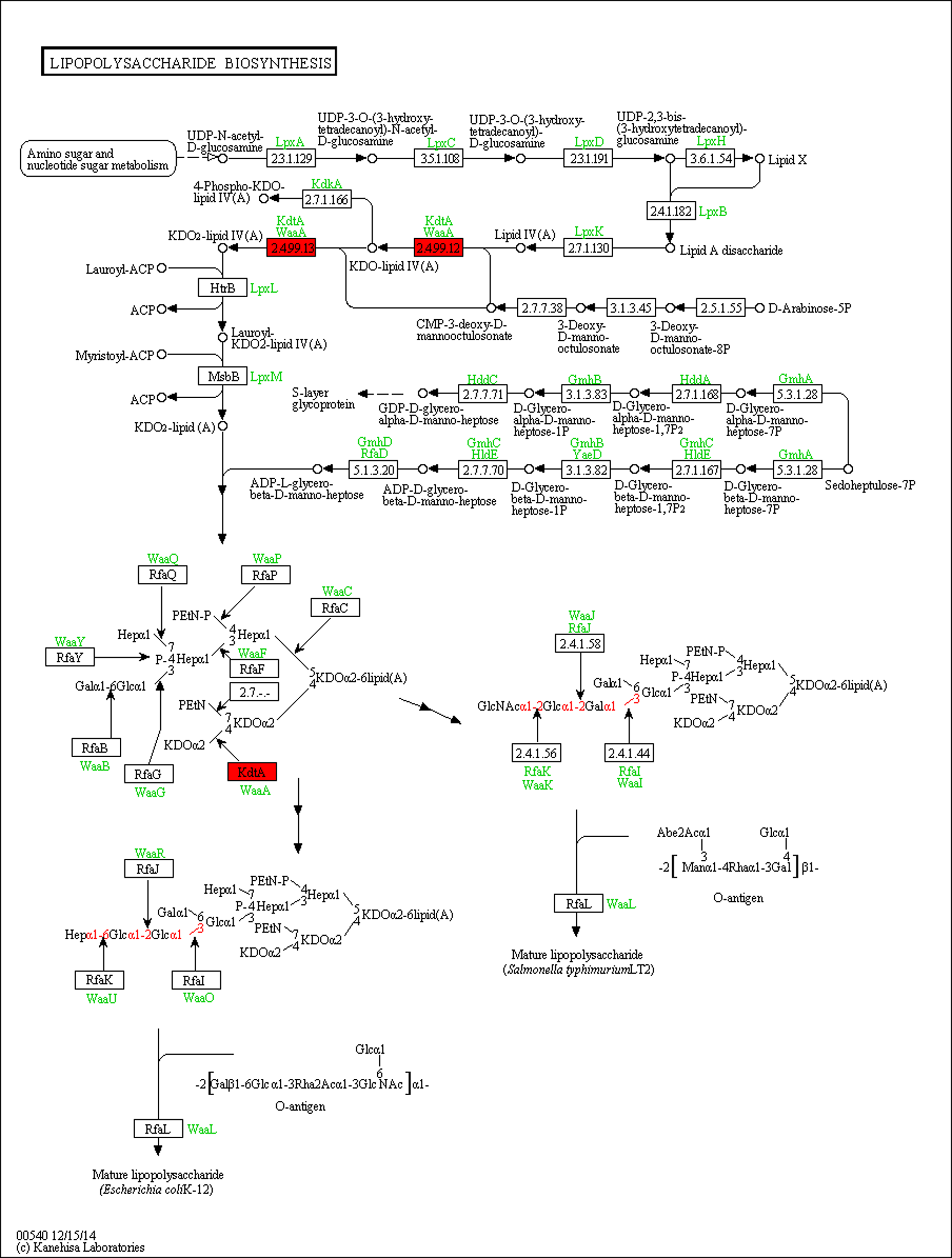

Supplement: Supplementary file 1 [file ijms-20-04227-s001.zip › ijms-559609-supplementary files/S1.tif]

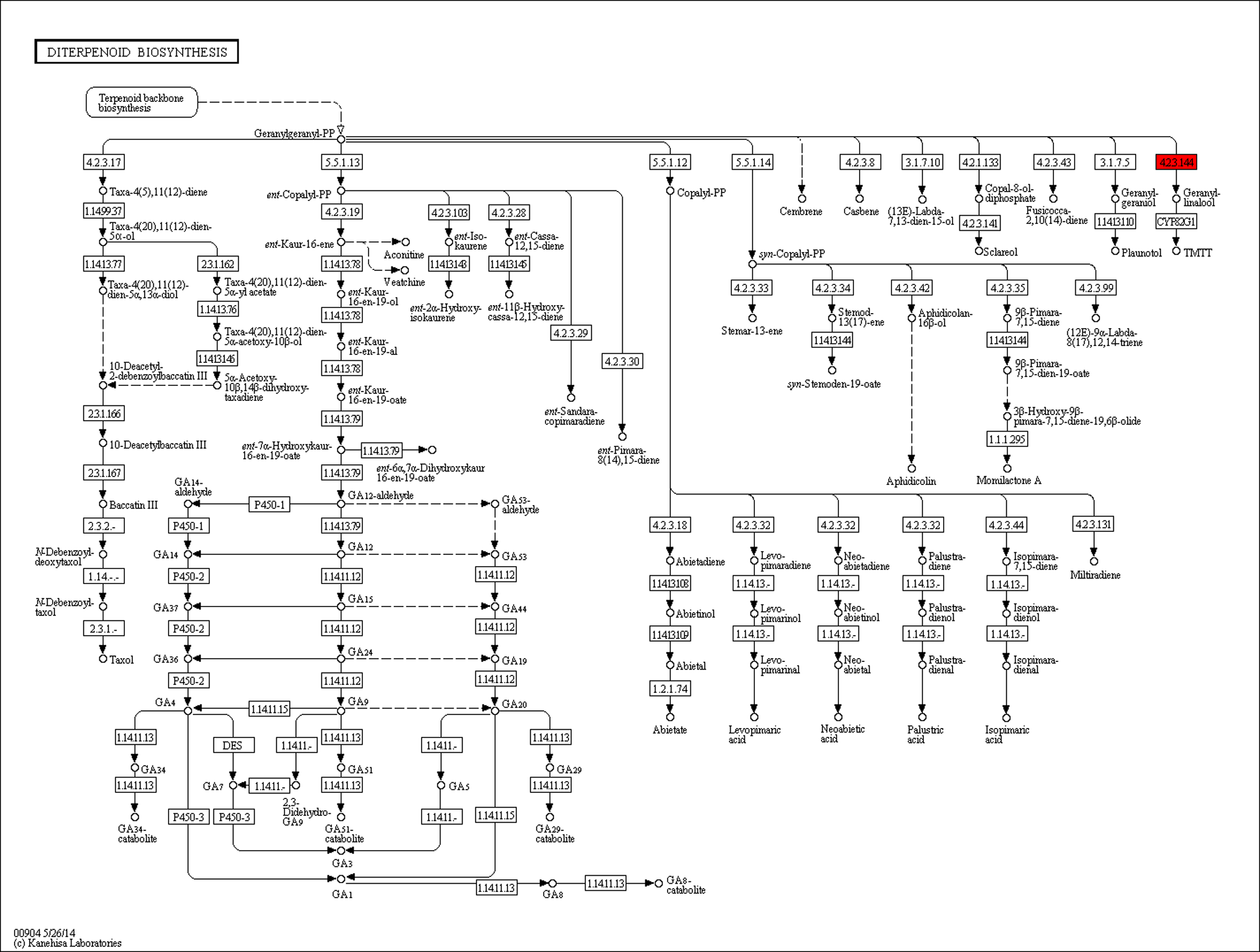

Supplement: Supplementary file 1 [file ijms-20-04227-s001.zip › ijms-559609-supplementary files/S2.tif]

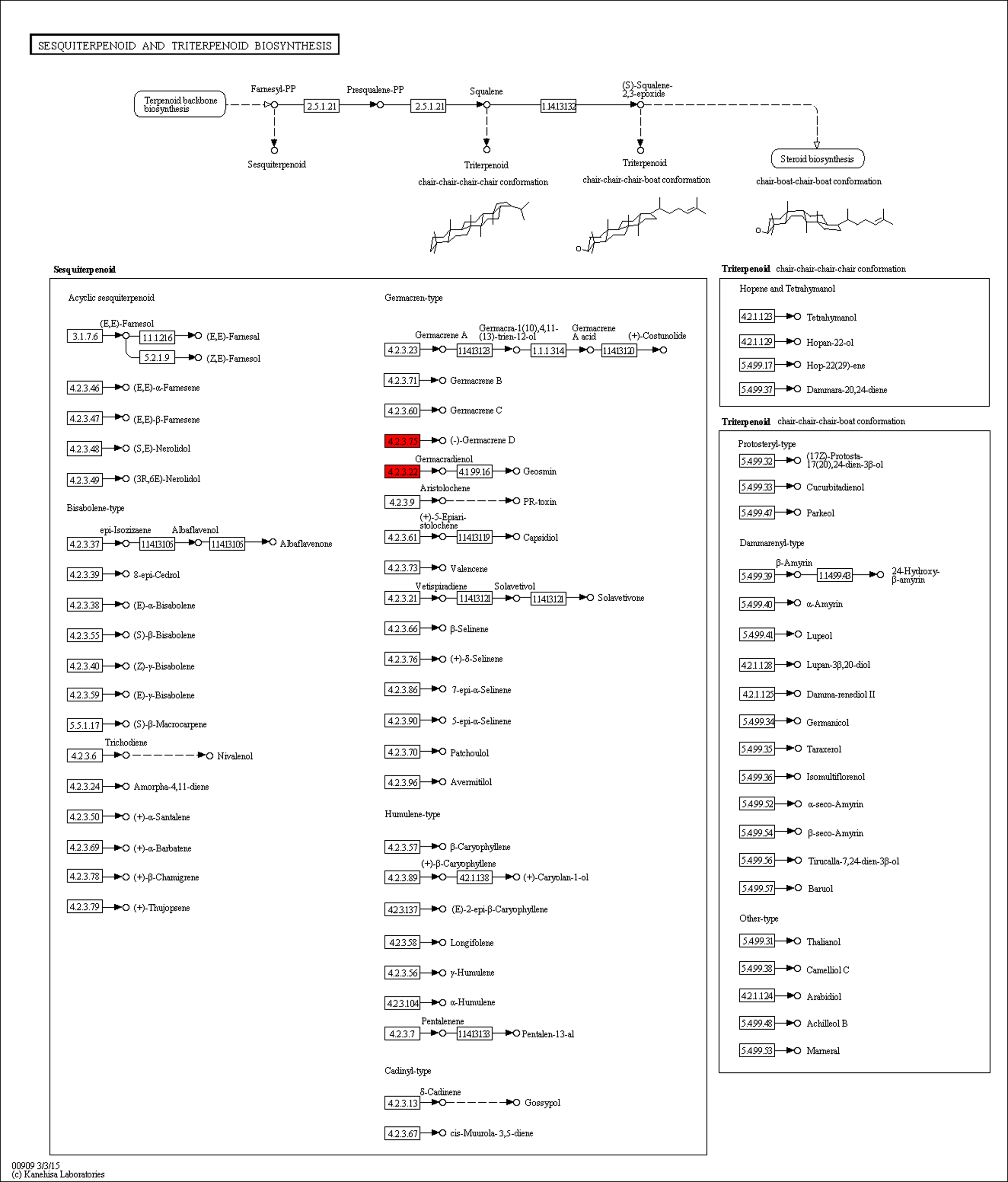

Supplement: Supplementary file 1 [file ijms-20-04227-s001.zip › ijms-559609-supplementary files/S3.tif]

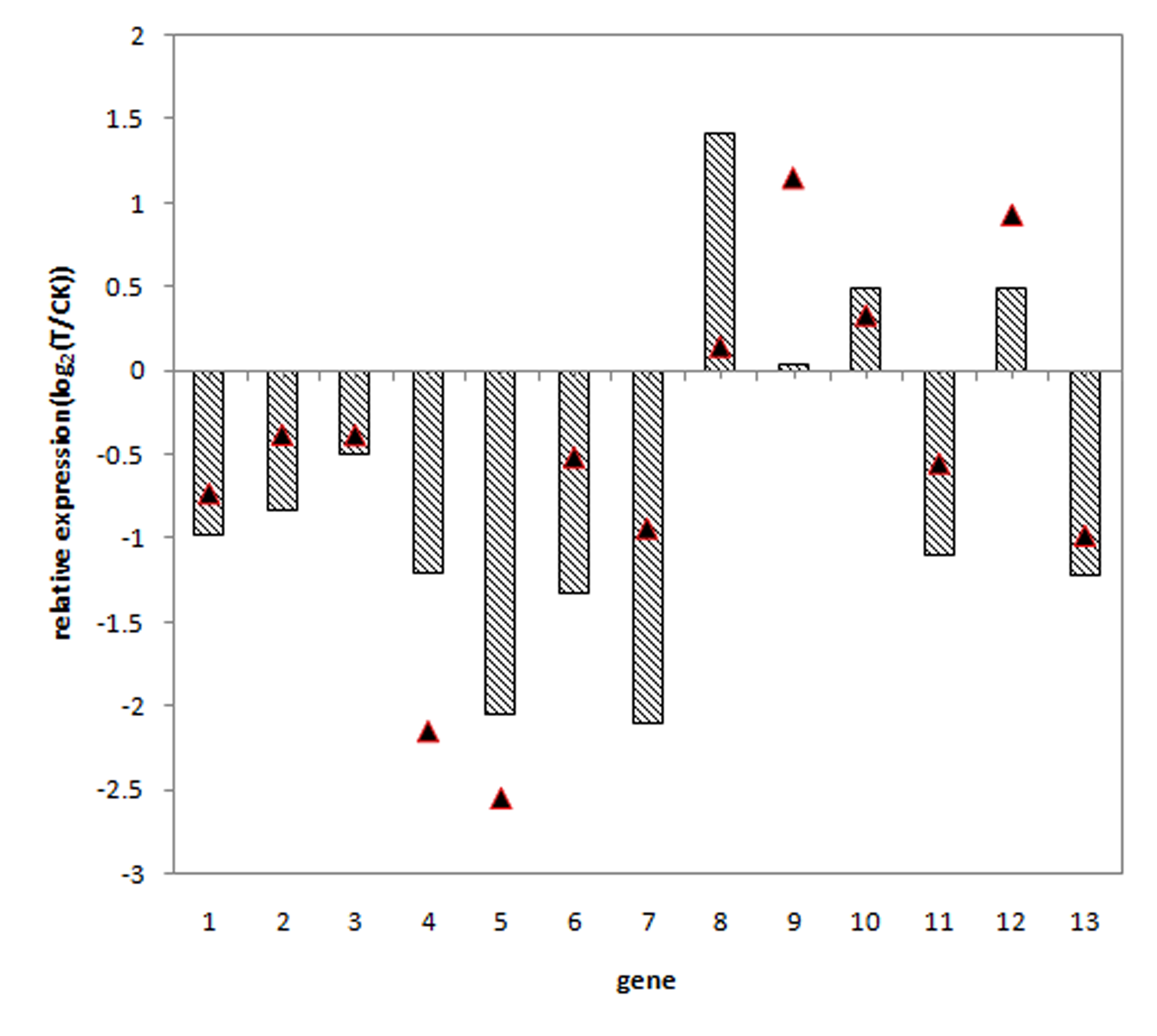

Supplement: Supplementary file 1 [file ijms-20-04227-s001.zip › ijms-559609-supplementary files/S4.tif]
